# Personalized therapy in rheumatoid arthritis (PETRA): a protocol for a randomized controlled trial to test the effect of a psychological intervention in rheumatoid arthritis

**DOI:** 10.1186/s13063-023-07707-0

**Published:** 2023-11-20

**Authors:** Lennart Seizer, Ellis Huber, Miriam Schirmer, Sven Hilbert, Eva-Maria Wiest, Christian Schubert

**Affiliations:** 1 Department of Child and Adolescent Psychiatry, Psychosomatics and Psychotherapy, University Hospital of Tübingen, Tübingen, Germany; 2 Institute of Psychology, University of Innsbruck, Innsbruck, Austria; 3 Department of Psychiatry, Psychotherapy, Psychosomatics and Medical Psychology, Medical University Innsbruck, Innsbruck, Austria; 4 Professional Association of Preventologists, Berlin, Germany; 5 Faculty of Human Sciences, University of Regensburg, Regensburg, Germany

**Keywords:** Psychoneuroimmunology, Randomized controlled trial, Integrative single-case study, Rheumatoid arthritis, Group-based intervention

## Abstract

Rheumatoid arthritis (RA) is a chronic autoimmune disease that primarily affects cartilage and bone. Psychological stress can both trigger disease exacerbation and result from disease activity. As standard pharmacological interventions alone have limited success in treating RA, a more comprehensive biopsychosocial approach to treatment has been recommended. In this prospective randomized controlled trial (RCT), a psychotherapeutically guided, group-based intervention program will be conducted with RA patients over a period of 9 months. This program combines a dynamic-interactional model with disorder-specific coping-oriented perspectives to improve patients’ social, emotional, and problem-solving competencies as well as stress system functional status. The enrolment of 440 patients, randomly allocated to either an intervention (*n* = 220) or control group (*n* = 220), is planned. To evaluate the intervention effect, various indicators of RA disease activity, stress system activity, and psychological condition will be assessed through sets of standardized questionnaires and biochemical analyses of blood and saliva samples. Moreover, healthcare-related costs for each patient will be obtained using routine health insurance data. Outcome variables will be measured in all patients at regular intervals prior to intervention (baseline), during the 9-month intervention (five time points), and during a 9-month follow-up phase (three time points), allowing the comprehensive analysis of within- and between-subject effects, i.e. trajectories of the target variables in the intervention and control groups. In addition, to investigate the intervention effects on real-life stress system functioning in RA, 10 integrative single-case studies (*n* = 5 from the intervention group, *n* = 5 from the control group) will be conducted. In each study, once before and after the 9-month intervention, urine samples will be collected, and patients will fill out questionnaires for approximately 1 month at 12-h intervals. Moreover, weekly in-depth interviews will be conducted with patients to determine their previous week’s emotionally positive and negative incidents. Using time series analysis, it is then possible to investigate whether and how stress system function in these RA patients has improved from the applied intervention. By using both an investigational macro- and microperspective, this project aims to evaluate a psychological intervention in the routine care of individuals with RA.

**Trial registration** German Clinical Trials Register DRKS00028144. Registered on 1 March 2022.

## Background

Rheumatoid arthritis (RA) is an autoimmune disorder of connective tissue that usually follows a chronic course with characteristic destruction of cartilage and bone but can also present extra-articular manifestations (e.g. skin, eyes, lungs, heart, blood vessels) [1]. The clinical onset of RA often manifests as inflammation in the synovium of the hand and finger joints. However, as the disease progresses, more joints throughout the body become affected leading patients to report high levels of disease burden due to pain and restrictions in movement [2]. Additionally, there is a high comorbidity with psychiatric disorders, with depression being the most commonly observed condition [3, 4]. Despite state-of-the-art pharmacological interventions in form of disease-modifying antirheumatic drugs (DMARDs), many RA patients still experience various physical and psychological symptoms [5]. This indicates the need for novel therapeutic and supportive strategies.

This is where the field of psychoneuroimmunology (PNI) becomes relevant. PNI integrates psychological, neuroendocrinological, and immunological factors in addressing health and disease. In addition to other influences (e.g. genetic aspects, chronic infections, sex hormones), psychosocial stressors have frequently been identified as a risk factor in RA, i.e. triggering the onset of the disease and the occurrence of flare-ups [6, 7] and negatively impacting adherence to treatment plans [8]. On the other hand, manifestations of RA disease activity (e.g. recurrent pain, fatigue, prolonged inflammation) themselves act as stressors and influence mental health via changes in cognitive appraisal, affective states, and behavioural coping responses. Several alterations in the stress system of RA patients have been found, e.g. a diminished adrenocorticotropic stress response [9, 10], a shift from β- to α-adrenoreceptors on the surface of immune cells [11–13], impairment in the inflammation-inhibiting capacity of cortisol [14], and a disturbed circadian rhythm [15]. These modifications could potentially result in increased pro-inflammatory responses and a deterioration in rheumatic activity.

In contrast, it has been shown that positive emotional experiences, such as mirthful laughter, were followed by changes in cytokine levels connected with a decrease in pro-inflammatory (TNF-α) levels and an increase in anti-inflammatory activity (IL-6, IL-4, IL-1RA) in RA patients [16]. Furthermore, in patients with systemic autoimmune disorders such as RA and systemic lupus erythematosus (SLE), use of the integrative single-case design - specifically developed for the naturalistic investigation of PNI mechanisms in health and disease - revealed a cyclic response pattern in urinary neopterin (an inflammation marker) following emotionally meaningful positive everyday incidents. This pattern ultimately resulted in decreases in neopterin levels after 3–4 days ([17], unpublished data). Based on these findings, it can be suggested that enhancing coping flexibility among RA patients and incorporating stress management programs into RA treatment may have a positive impact on the disease's outcome [18–21].

In a meta-review, Prothero et al. [18] analysed the effect of psychological interventions in patients with RA (8 reviews; 66 studies; 7279 patients) and found immediate postintervention improvements in various categories, namely, pain, fatigue, depression, anxiety, self-efficacy, coping behaviour, physical ability, and functional disability. These beneficial effects remained evident in follow-up assessments for depression (after 8.5 months), coping behaviour (after 8.5 months), self-efficacy (after 15 months), and physical activity (10–14 months). Disease activity and tender or swollen joints showed a delayed improvement after ten weeks and 8.5 months, possibly caused by mediating effects of the postintervention improvements (e.g. depression, coping). Additionally, it has been demonstrated that psychological interventions, including cognitive-behavioural pain management, emotional expression, and stress management, can influence the levels of stress system parameters associated with the immunopathogenesis of RA, such as IL-6, IFN-γ, cortisol, and IL-8 [19–21].

In this study, a psychotherapeutically guided, group-based intervention program will be conducted with a sample of RA patients over 9 months. This program synergistically combines a dynamic-interactional model with disorder-specific coping-oriented perspectives to improve participants’ social, emotional, and problem-solving competencies. The aim of the study is to evaluate the effect of the intervention program using multimodal data: multiple psychosocial and physiological (immunoendocrine) measurements, scores of RA disease activity, and healthcare-related data. Furthermore, it has been suggested that the complex relationship between a psychological intervention and changes in psychophysiological variables can be better investigated by combining both between- and within-subject data over an extended period [22]. Thus, for each patient, measurements of relevant variables will be conducted at regular intervals over 18 months: once before the intervention, five times during the 9-month intervention period, and three additional times during the 9-month follow-up phase. The control group will not participate in the intervention program, but measurements of variables will be performed according to the same temporal protocol, allowing the analysis of inter- and intrasubject variation.

In addition, integrative single-case studies will be performed on a subset of patients. Specifically, patients in both the treatment and control groups will be closely monitored over the course of approximately 1 month, with assessments taken at 12-h intervals in their everyday life (“life as it is lived”). These assessments will occur once before and once after the intervention and will include close-meshed biological (urine samples), psychological (questionnaires), and social (in-depth interviews) measurements [23]. The high ecological validity of the integrative single-case design with a focus on interview evaluation and analysis of biopsychosocial time series data will allow the real-life investigation of postinterventional changes regarding stress system functioning in RA. By doing so, potential PNI mechanisms mediating or moderating the effects of the intervention could be identified in an exploratory manner.

The project’s consortium includes the “Betriebskrankenkassen Landesverband (BKK-LV) Bayern” (Bavarian Association of Company Health Insurance Funds), responsible for study management and coordination; the “Allgemeine Ortskrankenkasse (AOK) Bayern” (General Local Health Insurance Fund Bavaria); the “Berufsverband der Präventologen” (Professional Association of Preventologists); the “Deutsche PsychotherapeutenVereinigung (DPtV) Landesgruppe Bayern” (Bavarian regional group of the German Psychotherapists Association); the “Berufsverband Deutscher Rheumatologen (BDRh)” (Professional Association of German Rheumatologists); the “Kassenärztliche Vereinigung Bayerns (KVB)” (Bavarian Association of Statutory Health Insurance Physicians); the “Universität Regensburg (UR)” (University of Regensburg); and the “Medizinische Universität Innsbruck (MUI)” (Medical University of Innsbruck). The consortium members do not declare any financial or other competing interests. The project is publicly funded by the “Gemeinsamer Bundesausschuss (G-BA)” (Innovation Committee at the Federal Joint Committee) (grant number: 01NVF20024). The funding agency did not have any role in creating the study design and will not be involved in collecting, managing, analysing, or interpreting data. The study protocol and other relevant study documents were approved by the ethics commissions of the UR (21–2604-101) and the “Bayerische Landesärztekammer (BLÄK)” (Bavarian Medical Association) (19005); the study is registered with the German Clinical Trials Register (DRKS00028144). Protocol modifications will be communicated to the responsible ethics commission and the trial registry. Written, informed consent for participation will be obtained from all participants.

## Methods

### Study design

This study evaluates the effect of a self-management education program on several indicators of disease activity and associated psychosocial and physiological variables in patients diagnosed with RA and will take place in Bavaria, Germany, from March 2022 to December 2024. Enrolled patients (*n* = 440) will be randomly assigned (randomized) in equal numbers to either an intervention (*n* = 220) or control group (*n* = 220). The intervention group will participate in 15 2-h sessions over nine months in subgroups of 6–12 participants, aimed at enhancing individual health and life skills. The program will be led by psychotherapists who are specifically trained.

**Fig. 1:**
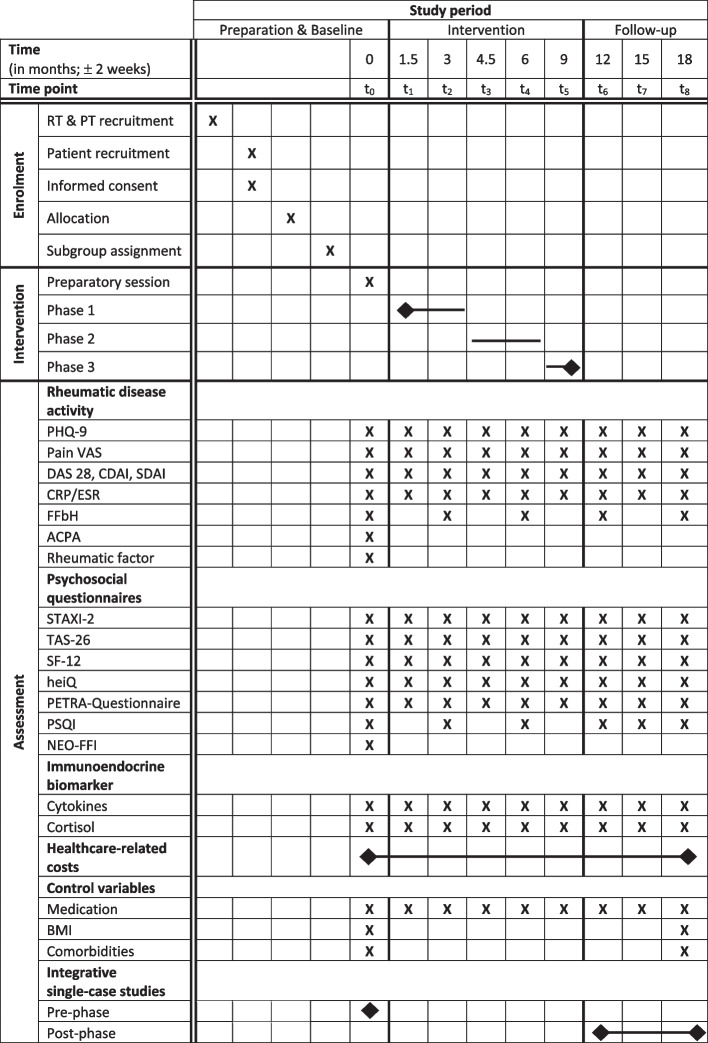
Project timeline RT, rheumatologist; PT, psychotherapist; PHQ-9, Patient Health Questionnaire; VAS, visual analogue scale; DAS 28, Disease Activity Score; SDAI, Simplified Disease Activity Score; CDAI, Clinical Disease Activity Score; FFbH, Funktionsfragebogen Hannover; CRP, C-reactive protein; ESR, erythrocyte sedimentation rate; ACPA, anti-citrullinated protein antibody; STAXI-2, State-Trait Anger Expression Inventory-2; TAS-26, Toronto Alexithymia Scale; SF-12, 12-item Short Form Survey; heiQ, Health Education Impact Questionnaire; PSQI, Pittsburgh Sleep Quality Index; NEO-FFI, NEO Five-Factor Inventory; BMI, body mass index; Cytokines, TNF-α, IL-1β, IL-2, IL-2R, IL-6, IL-10, IL-12, IL-13

The training subgroups will not run simultaneously but will start independently whenever enough patients in a region have enrolled to form an intervention subgroup. To evaluate the effect of this intervention, various indicators of RA disease activity, immune and stress system activity, and psychological condition will be assessed through a set of standardized questionnaires and biochemical analyses of blood, urine, and saliva samples. Outcome variables will be measured at regular intervals before the intervention (baseline), during the 9-month intervention (five time points), and during the 9-month follow-up phase (three time points) at patients’ appointments with their respective rheumatologists. Further data on health-related costs will be acquired through the participating insurance companies (BKK-LV Bayern and AOK Bayern). The subsequent statistical analysis will then assess intra- and interindividual effects based on comparing time points and study groups. Furthermore, a total of ten patients (*n* = 5 from the intervention group, *n* = 5 from the control group) will be investigated thoroughly in integrative single-case studies [21] using biopsychosocial measurements collected at 12-h intervals to produce time-series data for approximately 1 month before and after the intervention. Patients allocated to the control group will receive an expense allowance of 260 Euros to cover the 26 h required for project-related activities during the nine time points of the study (equivalent to ten euros per hour). All patients will continue to receive standard treatment from their rheumatologists during the study. An overview of the study’s timeline can be seen in Fig. 1. No adverse events or harm is expected from the study.

### Population, recruitment, and allocation

The study population will consist of patients diagnosed with seropositive chronic polyarthritis (ICD-10-Code M05) or another chronic polyarthritis (ICD-10-Code M06) with a minimum age of 18, who are being treated by an eligible rheumatologist in Bavaria and are insured with either BKK-LV Bayern or AOK Bayern. Furthermore, participants need to have sufficient German language skills to complete the questionnaires used by the study. Patients with a diagnosis of fibromyalgia (ICD-10-Code M79.7) will be excluded from the study. The probands will primarily be recruited with the assistance of rheumatologists who practice in the study region and will introduce the study to their patients. Furthermore, the study will be promoted in relevant self-help networks, and some patients who gave prior consent to receive such offers will get a postal notification by BKK-LV Bayern or AOK Bayern. The goal is to enrol 440 probands, who will be randomized in equal proportions into the intervention and control groups (each *n* = 220). Based on simulations using the *lme4* package in *R* [24, 25], this sample should allow the identification of even small effects (*f* = 0.1) with adequate statistical power (1 – *β* > 0.8) while considering a possible drop-out rate of 20%.

Prior to patient enrolment, eligible registered rheumatologists in Bavaria will be contacted and asked to participate in the study. After application and confirmation of participation, the rheumatologists will receive comprehensive instructions on their tasks and responsibilities within the study. In addition, psychotherapists can apply to lead an intervention subgroup. Before the study starts, psychotherapists will be selected from the applicants using an assessment process and receive comprehensive training in the content, background, and implementation of the PETRA intervention program (“train the trainer”).

### Intervention

The intervention is a psychotherapeutically guided, psychoeducational and group-based program designed to strengthen individual health and life skills. It was developed with the help of experienced psychological and medical psychotherapists and aims to improve the emotional, social, and problem-solving skills of each participant by synergistically combining a dynamic-interactional model with disorder-specific coping-oriented perspectives and integrating concepts, ideas, and experiences of existing self-management programs (e.g. the “Gesundheits- und Lebenskompetenz Training [GLK]”, the “HEDE [Health-Ease and Dis-Ease]-Training”, the “Bochumer Gesundheitstraining”, the “Hildesheimer Gesundheitstraining [HGT]”, or the “Zürcher Ressourcen Modell [ZRM]”) [26]. The intervention will be conducted in subgroups of 6–12 participants and led by psychotherapists trained explicitly for this purpose. The main phase consists of 12 2-h sessions over 6 months that address relevant health-associated topics and are conducted at biweekly intervals. Three additional 2-h sessions will take place at monthly intervals, in which the participants can jointly reflect on previous changes and consolidate a viable future perspective for improving their ability to cope with the disease. Each session will begin with an orientation round during which patients check in and prepare for the session. It will end with a closing round where patients can reflect on and share their experiences and insights from the session. This process helps to clarify possible misunderstandings and address any disappointments promptly.

Before the actual start of each 12 session intervention program, there will be a preliminary one-on-one meeting (approximately 45 min) with each participant conducted by the psychotherapist leading the group so they can familiarize with each other, clarify intervention expectations, and discuss possible intervention fears. Then, if necessary, there can be a second meeting.

The first phase of the intervention program (information phase) comprises six group sessions over 3 months at 2-week intervals, each lasting approximately 2 h. During the first two sessions, the objectives are to get to know each other, build mutual trust, and encourage confidence within the group. Furthermore, the program will be explained, and each patient will voice his or her personal health goals and expectations for the program. The subsequent four sessions (3–6) will address health-associated topics relevant to RA, e.g. nutrition, physical activity, sleep, and stress. Therefore, basic knowledge of each of the topics and its relevance to RA will be shared and subsequently deepened upon using a variety of group exercises, e.g. self-reflective or interactional. In this phase, patients will develop a biopsychosocial understanding of health and disease. This involves recognizing connections between psychological factors (e.g. stress, conflicts, persistent worries) and physical processes, and learning about possible ways to influence and personalize disease management.

The second phase of the intervention (open phase) will also last 3 months with six 2-h group sessions at 2-week intervals. During sessions 7–11, the psychotherapists will refrain from specifying the content of the discussion. Instead, participants will be actively encouraged to express their concerns, such as coping with stress or specific conflicts, and to receive support from the group. The aim of these sessions is to help patients identify personal resources and improve individual disease management, emotion regulation, and conviction of self-efficacy. In addition to the orientation and closing rounds, the structure of each session can vary depending on the topics introduced and discussed by participants and developing group dynamics. Finally, in the last session (12), the group will prepare for the end of the main phase of the program and reflect on progress and improvements.

The third phase of the intervention (sustainability phase) will also last 3 months but contains only three 2-h group sessions at monthly intervals. The psychotherapists will take a more active, leading role again and prepare the patients for detachment from the group and the transition to everyday life without the group. In the last session (15), the group members will reflect and give feedback on the program.

### Outcome measures

Measurements of the target variables and additional data will be collected at multiple time points throughout the study period by participating rheumatologists and psychotherapists (Fig. 1). The first measurement time will be a maximum of 2 weeks prior to the intervention as a baseline (*t*_0_). Then, five more measurements will be taken every 6 weeks during the 9 months of intervention (*t*_1_: 1.5 months after intervention start, *t*_2_: after 3 months, *t*_3_: after 4.5 months, *t*_4_: after 6 months, *t*_5_: after 9 months) and three more measurements will be taken every 3 months during the follow-up phase (*t*_6_: after 12 months, *t*_7_: after 15 months, *t*_8_: after 18 months). The time points will have a temporal range of ± 2 weeks for each proband, depending on each participant’s appointment at the rheumatologist’s office.

The psychological condition of each participant will be determined with a set of questionnaires. The State-Trait Anger Expression Inventory-2 (STAXI-2) is a questionnaire that measures various aspects of anger as an emotional state (state anger) and the disposition to experience angry feelings as a personality trait (trait anger) [27]. The Toronto Alexithymia Scale (TAS-26) assesses difficulties in identifying and describing feelings or emotions [28]. The 12-item Short Form Survey (SF-12) captures the general physical and mental health status and its implications for an individual’s everyday life [29].

The Health Education Impact Questionnaire (heiQ) measures the effectiveness of the group intervention related to the proximal outcome criteria of compliance, self-efficacy, and health behaviours, collectively understood as health literacy [30]. The Pittsburgh Sleep Quality Index (PSQI) assesses sleep quality, sleep disturbance, and possible sleep disorders [31]. The Depression Module of the Patient Health Questionnaire (PHQ-9) measures the severity of depression by scoring all nine DSM-IV depression criteria [32]. Last, the PETRA questionnaire was developed to be complementary to the other questionnaires to assess possible influencing factors on the course of the disease, i.e. current psychotherapeutic treatment, life events, physical activity and relaxation exercises. These questionnaires will be administered at all time points (*t*_0_–*t*_8_) during the rheumatologist appointments, except for the PSQI, which will be distributed only every 3 months (*t*_0_, *t*_2_, *t*_4_, and *t*_6_–*t*_8_). At the beginning of the intervention, patients in the intervention group will also complete the NEO Five-Factor Inventory (NEO-FFI), which evaluates the five domains of personality: neuroticism, extraversion, openness, agreeableness, and conscientiousness [33].

Physiological measurements will be assessed in blood and saliva samples, which the rheumatologists will collect at each appointment from the patients. Several cytokines and associated parameters, namely, tumour necrosis factor α (TNF-α), interleukin-1β (IL-1β), interleukin-2 (IL-2), interleukin-2 receptor (IL-2R), interleukin-6 (IL-6), interleukin-10 (IL-10), interleukin-12 (IL-12), interleukin-13 (IL-13), C-reactive protein (CRP), and erythrocyte sedimentation rate (ESR), will be analysed in plasma samples to assess immune system activity. Furthermore, cortisol levels will be measured in saliva samples. Cytokine and cortisol concentrations will be assessed at all time points. Additionally, at *t*_0_, anti-citrullinated protein antibody (ACPA) and IgG Fc autoantibody (rheumatic factor) levels will be measured. The rheumatologists are responsible for sampling and preprocessing all specimens and will determine ACPA, rheumatic factor, and CRP/ESR levels at *t*_0_, *t*_1_, *t*_3_, and *t*_5_–*t*_8_ within the frame of standard patient care. An external professional laboratory will be engaged to perform all other biochemical analyses in this study. For shipment from the rheumatologists to the laboratory, blood samples will be centrifuged (3000 rpm for 10 min) in serum gel tubes and subsequently stored at − 20 °C, and saliva samples will be collected using a cotton swab and shipped at room temperature.

RA disease activity will be measured using the Disease Activity Score 28 (DAS 28), the Simplified Disease Activity Index (SDAI), and the Clinical Disease Activity Index (CDAI) [34]. These composite scores are standard in clinical practice and based on questions about rheumatic pain, swelling, and tenderness of joints evaluated by the patient and the attending physician, combined with levels of CRP/ESR. Additionally, the Hannover Functional Ability Questionnaire (FFbH) will be conducted to assess functional capacity and impairment during rheumatic patients’ daily activities [35]; the patients rate their overall pain on a 10 cm visual analogue scale (VAS). The DAS 28, SDAI, CDAI, and pain VAS will be assessed at all measurement time points by the rheumatologists; the FFbH will be administered at every second time point (*t*_0_, *t*_2_, *t*_4_, *t*_6_, and *t*_8_).

Healthcare-related costs for each patient will be assessed using routine health insurance data. For this purpose, the number and type of all physician visits, supplementary therapies, drug costs, the number and duration of hospital stays, days of incapacity to work and the resulting inpatient, outpatient, and total health insurance expenditures over the 18-month study period (from *t*_0_ to *t*_8_) will be obtained from the respective healthcare provider.

Patients from the intervention group and each psychotherapist leading a group will evaluate their perception and experience of the training and provide this information via newly developed questionnaires at the beginning and end of the intervention program.

Additionally, BMI, concomitant diseases, and rheumatic medication intake will be assessed as control variables. Height, weight, and concomitant diseases will be captured at *t*_0_ and *t*_8_, and the rheumatologists will provide the medication regimen at each time point. The following types of medication will be considered: cortisone preparations, conventional synthetic or targeted synthetic disease-modifying antirheumatic drugs (cs/tsDMARD), and biological or biosimilar disease-modifying antirheumatic drugs (b/bsDMARD).

### Integrative single-case studies

A subset of ten patients (five from the intervention group and five from the control group) will be monitored closely before and after the intervention phase in the form of integrative single-case studies performed by the MUI [23]. Shortly before the start of each of these studies, a patient will undergo a thorough physical exam by his or her rheumatologist. Moreover, interviews (e.g. Structured Clinical Interview for DSM-5 [SCID-5], Life Events and Difficulties Schedule [LEDS], Operationalized Psychodynamic Diagnosis [OPD]) will be conducted in person with patients for the following purposes: (1) psychiatric evaluation, (2) recording the previous 2 years’ life events and difficulties, (3) obtaining in-depth information about the patient’s biopsychosocial biography, and (4) getting to know patients and establishing a trusting relationship for the upcoming weekly interviews.

During the data sampling period of the integrative single-case studies, patients will collect all of their urine in 12-h intervals (~8 am to ~8 pm and ~8 pm to ~8 am) for a period of approximately 1 month. Upon collection, Na-metabisulfite and Na-EDTA will be added to the urine canisters to prevent sedimentation and oxidation. After collection and aliquotation, samples will be stored at − 20 °C at home for the study duration and afterwards at − 80 °C in the clinic until further analysis of various stress system and immune parameters such as urinary cortisol, neopterin, and IL-6.

Furthermore, patients will retrospectively complete a set of questionnaires at the end of each 12-h interval, i.e. at ~8 am and ~8 pm. Specifically, using an adapted version of the Daily Inventory of Activity, Routine and Illness (DIARI), the patient will answer questions about RA symptoms, daily lifestyle factors (e.g. physical activity, alcohol consumption, medication, sleep behaviour), psychological factors (e.g. emotional states, emotional competence, social support), and potential signs of infection. Moreover, the patient will take notes on all psychosocial incidents (positive and negative) during the preceding 12 h.

Furthermore, weekly in-depth psychological interviews (lasting approximately 1 h) using the Incidents and Hassles Inventory (IHI) will be conducted with the patient via online videoconferences. These interviews aim to identify the previous week's negative and positive emotionally meaningful incidents, which will subsequently be rated by a three-person panel following the study period. Additionally, the patients will have weekly appointments with their rheumatologists to determine their DAS 28 (CRP/ESR), SDAI, and CDAI scores.

Using the integrative single-case design, researchers can construct various equidistant time series from biological, psychological, and social datasets. These time series can be statistically analysed to determine their temporal relationships. For instance, one can investigate whether one variable significantly precedes or follows another [36, 37], identify response patterns between variables [17, 38], and determine the duration of temporal delays between variables [39, 40]. For further details on the study design, see [23].

Before the intervention begins, all ten patients participating in the integrative single-case studies will collect data as previously described for the 3-month period leading up to the intervention. In contrast, the follow-up data collection periods are staggered: two patients will be monitored within 1 to 4 months after the intervention ends, four patients within 5 to 8 months after, and four more patients within 9 to 12 months after, each for approximately 1 month. This design allows for the identification of functional changes before and after the intervention, such as alterations in the patients’ psychophysiological response to emotionally meaningful positive and negative incidents.

### Study procedures and data management

Patients who want to participate in the study will receive informational materials from their respective (participating) rheumatologist, which contains a comprehensive and understandable study description. The consent form (for the study in general and the integrative single-case studies specifically) will also be provided by the rheumatologist, who will pass it on either to BKK-LV Bayern or AOK Bayern (dependent on the patient’s insurance) after the patient signed it. Patients must provide written, informed consent before any study procedures can occur. Participating patients will indicate their consent by signing a form in which they agree to the use of their data for the specific purposes. Only data pertinent to project implementation and scientific monitoring will be gathered, processed, and utilized. Patients have the right to withdraw their consent at any time without needing to provide reasons.

The data protection concept follows the principle that at no time will clear names and patient data be present in a dataset. For this purpose, all patients will be given pseudonyms on the basis of a randomization list that has been created by the UR. Collection, merging, evaluation, and storage of the data in paper form will be carried out by different institutions. Participating patients will be randomly assigned into an intervention group or a control group. The BKK-LV Bayern and the AOK Bayern will randomize the enrolled patients to the intervention or control group based on previously created pseudonymized participant IDs. Within the project consortium, conclusions about the person will be possible only for the BKK-LV Bayern and the AOK Bayern, as well as for the patient controller with the help of the key table. The blank randomization lists will be password-protected, encrypted and stored in a secure FTPS directory.

After enrolment, the patients will be randomly allocated to the intervention or control group in a 1:1 ratio, alternately dependent on the time of registration, using a prefabricated list of pseudonymous six-digit proband IDs created using the *random*-package in Python [41]. These IDs will be used to combine data from different sources for evaluation purposes pseudonymously. The UR will be responsible for evaluating study data and monitoring the project progress, whereby data from different sources (RheMIT, archive service provider, laboratory, BKK-LV Bayern, AOK Bayern) will be combined and sorted according to the proband IDs. If missing data or unattended appointments are found, the UR will contact respective responsibilities to evaluate further procedures.

A designated proband guide will handle a large part of the communication and information flow towards patients, rheumatologists, and psychotherapists. The BKK-LV Bayern and AOK Bayern will inform the proband guide about each enrolled patient, who will then inform the patients about their group allocation and patients in the intervention group about possible intervention subgroups near them. When 6–12 participants are available for a subgroup, the proband guide will close admissions for that subgroup, set an intervention start date, and provide the participants' contact information and questionnaire copies (NEO-FFI, anamnesis and evaluation form) to the assigned psychotherapist. Each psychotherapist leading a subgroup will track the attendance of participants at each session and transmit lists to an archive service provider, who will forward these to the UR.

Furthermore, the proband guide will inform the rheumatologists of the patients in each subgroup so that they can arrange appointments at the measurement time points (*t*_0_–*t*_8_). For secure exports of datasets to the study's evaluators, some of the measurements that are assessed in these appointments (CRP/ESR, SDAI, CDAI, DAS 28, VAS, PHQ9, FFbH, PSQI, BMI, medication, comorbidities, rheumatic factor levels, ACPA levels) will be documented by the rheumatologists using standardized forms in particular software (RheMIT | BDRh Service GmbH [bdrh-service.de]). The UR will receive the pseudonymized (proband ID) data for all patients, while the MUI will receive only data for the integrative single-case study patients. Other questionnaires, which will also be administered at the appointments and cannot be documented in RheMIT (STAXI-2, TAS-26, SF-12, heiQ, PETRA-Questionnaire), and the questionnaires, which will be completed during the intervention’s group sessions with the psychotherapist, will be mailed by the rheumatologists and psychotherapists to an archive service provider, who will digitalize the data and forward it to the UR. Data from the integrative single-case study participants will then be sent to the MUI by the UR. The data on healthcare-related costs will be pseudonymized and sent directly to the UR by BKK-LV Bayern and AOK Bayern.

For physiological measurements performed by the external professional laboratory, the rheumatologists will send preprocessed samples (blood and saliva) to the laboratory, where the mentioned biochemical analyses will be performed, and the pseudonymized results will be transmitted to the UR. In the integrative single-case studies, laboratory equipment necessary for urine sampling and storage and all questionnaires will be provided to the patients before the data sampling period starts and will be picked up afterwards by the MUI. The MUI will perform subsequent biochemical analyses of urine samples.

All patient data transfers will be performed securely, either by mail or encrypted email. The data flow architecture and study procedures have been reviewed and approved by the Bavarian state commissioner for data protection. In this study, a data monitoring committee has not been established. To ensure that the data from the various sources are transferred securely to the UR (evaluating institution), a server will be rented from the company Pegasus GmbH. Pegasus GmbH will assume the role of security management and manage server hosting. Pegasus GmbH is ISO/IEC 27001 and ISO/IEC 27018 certified. The servers are in Germany. Before the exchange, all files to be transmitted will be encrypted. For this purpose, asynchronous encryption is used. Each partner, similar to the UR, creates a private and a public key, and they exchange these keys among one another. The files are signed using the partner’s private key so that the recipient can verify the sender’s source, and the files are encrypted using the partner’s public key so that only the owner of the key counterpart can decrypt them. The study design is open-label with only outcome assessors being blinded, so unblinding will not occur.

### Statistical analyses

A central question in the statistical analyses represents the interaction of the intersubject factor group membership (intervention or control group) and the intrasubject factor time of measurement, i.e. the question of the target variables’ different trajectories in the intervention and control group. For longitudinal data analysis, generalized linear mixed models will be estimated, and due to their flexibility, they can model both normally distributed data and count data as dependent variables. In addition, missing values due to drop-outs are less of a problem for mixed longitudinal models compared to analyses of variance, providing a major advantage in analysing clinical programs. For all outcome variables, covariates will be included in the analyses as potential predictors (e.g. age, personality, gender, the severity of illness at baseline).

In the context of these generalized linear mixed models, quasi-Poisson regressions are the preferred method for modelling healthcare-related costs, which are associated with the predictors through log-link functions. Quasi-Poisson regression is preferred over classic Poisson regression for this project because it also allows modelling overdispersion in count data through an additional variance parameter. Immunoendocrine marker levels, questionnaire data and healthcare-related data with an approximated normal distribution, in turn, can be modelled linearly or estimated according to the divergent distribution with a fitted link function. Comparisons between the intervention and control groups will be directly integrated into the mixed regression models with a dichotomous indicator variable, whereby the influence of other covariates in the comparison can be accounted for. Statistical significance is indicated by a *p* value < 0.05. In addition, the Benjamini–Hochberg method will be applied as a correction procedure to avoid the inflation of type I errors in multiple analyses.

In the statistical analyses of the integrative single-case studies, time series variables will be tested on mutual dependency using autoregressive integrated moving average models (ARIMA), cross-correlational analyses and vector autoregressive models (VAR). First, it will be necessary to consider that each time series––psychosocial or biochemical––is governed by two main factors: an internal dynamic structure, giving rise to serial dependency, and serially uncorrelated disturbances. Because of such serial dependencies, an unadjusted cross-correlational analysis may lead to spurious correlations. Therefore, these dependencies will be separated from the disturbances by applying ARIMA filters to the time series. To determine whether one variable significantly precedes and thus predicts another, cross-correlations with the filtered residuals will be computed, both at lag zero (i.e. contemporaneous correlation) and higher lags (up to ± 14) with a significance level of *p* < 0.05.

All planned analyses will be performed with both the newest version of IBM SPSS Forecasting [42] and statistical software *R* [25] using the packages *lme4* (generalized linear mixed-effects models) [24], *tseries* (basic time-series analysis) [43], *forecast* (ARIMA models) [44], *vars* (VAR models) [45], and *ggplot2* (graphical processing) [46].

## Discussion

RA has numerous implications. It affects individuals, causing chronic pain, fatigue, insomnia, and limitations in performing daily activities. At the public health level, it generates a population of patients with lasting disabilities who require long-term therapy. This study adopts a holistic treatment approach that integrates various disease aspects, considering their interactive relations in accordance with the biopsychosocial model [47]. RA has been demonstrated to impact not only physiological and psychological outcomes but also to emerge or worsen due to psychophysiological mechanisms triggered by psychosocial stressors. A combination of different treatment modalities may prove more efficient over time at the individual and public health levels by improving the RA disease activity of the patients, increasing their quality of life and reducing treatment expenditures.

Methodologically, the proposed PNI project consists of a combination of two main study approaches, which both integrate biopsychosocial datasets collected over time with temporal data analyses, allowing a comprehensive analysis of within- and between-subject effects. This represents an expansion and innovation compared with the usual RCT approach to the topic under study [3]. Especially with our second approach, integrative single-case studies, it becomes possible to move beyond simply determining a treatment effect achieved by pooling data across participants and instead examine *interindividual* variation. Rather, this approach takes a “microscopic” perspective by analysing the *intraindividual* variation of real-life biological, psychological, and social data over time, thereby investigating under the highest possible ecologically valid conditions (“life as it is lived”) *how* an intervention changes the functional characteristics of the stress system in individuals with RA.

This study has all of the usual limitations inherent to any study with a limited sample size and data acquired in a limited region and period in terms of generalizability. In addition, other biases could emerge from patients’ voluntary and self-motivated participation after receiving information about the study goals, which could selectively favour the enrolment of patients who are receptive to psychological intervention. Furthermore, this study aims to evaluate the benefits of the psychological intervention as an addition to treatment-as-usual with a waiting-list control. Therefore, it will only provide limited insights into the mechanisms of effects that could be tested with additional (active) control groups.

The proposed study is clinically and methodologically in line with the biopsychosocial model [47, 48], a paradigm in medicine that focuses on the complex and thereby individual aspects of human life. In this regard, personalized and individual health literacy concepts represent an innovation that promotes individual health and life skills, enables patients to experience self-efficacy, and effectively strengthens their self-management. Improving health literacy among groups incurring high healthcare costs will yield significant benefits for both the affected individuals and the healthcare systems.

We plan to disseminate the study results in relevant peer-reviewed journals and conferences. The decision-making authority on publishing details lies within the project’s consortium. Furthermore, to provide flexible answers to research questions and follow-up investigations, a second dataset that does not include any patient-identifying variables or routine data from the health insurance companies will be created in addition to the primary dataset. As a result, the dataset will be completely anonymized and can be forwarded for the purposes mentioned above.

## Trial status

(Protocol version 5.0, 24th October 2023)

Recruiting started on 1st February 2022 and ended on 30th September 2023. The study successfully enrolled a total of 73 individuals, with 34 participants in the control group (2 withdrawals) and 39 participants in the intervention group (8 withdrawals). As of now, one integrative single-case study has been conducted. Additional integrative single-case studies are pursued.

## Data Availability

The datasets analysed during the current study and the statistical code are available from the corresponding author upon a reasonable request, as is the full protocol. The participant informational materials and informed consent form are available from the corresponding author upon request.

## Abbreviations

RA: Rheumatoid arthritis
EULAR: European League Against Rheumatism
BKK-LV Bayern: Betriebskrankenkassen Landesverband Bayern (Bavarian Association of Company Health Insurance Funds)
AOK Bayern: Allgemeine Ortskrankenkasse Bayern (General Local Health Insurance Fund Bavaria)
DPtV Landesgruppe Bayern: Deutsche PsychotherapeutenVereinigung Landesgruppe Bayern (Bavarian regional group of the German Psychotherapists Association)
BDRh: Berufsverband Deutscher Rheumatologen (Professional Association of German Rheumatologists)
KVB: Kassenärztliche Vereinigung Bayerns (Bavarian Association of Statutory Health Insurance Physicians)
UR: Universität Regensburg (University of Regensburg)
MUI: Medizinische Universität Innsbruck (Medical University of Innsbruck)
G-BA: Gemeinsamer Bundesausschuss (Innovation Committee at the Federal Joint Committee)
BLÄK: Bayerische Landesärztekammer (Bavarian Medical Association)
ICD-10: International Statistical Classification of Diseases and Related Health Problems
GLK: Gesundheits- und Lebenskompetenz Training
HEDE: Health-Ease and Dis-Ease
HGT: Hildesheimer Gesundheitstraining
ZRM: Zürcher Ressourcen Modell
STAXI-2: State-Trait Anger Expression Inventory-2
TAS-26: Toronto Alexithymia Scale
SF-12: 12-Item Short Form Survey
heiQ: Health Education Impact Questionnaire
PSQI: Pittsburgh Sleep Quality Index
PHQ-9: Depression Module of the Patient Health Questionnaire
DSM-IV: Diagnostic and Statistical Manual of Mental Disorders
NEO-FFI: NEO Five-Factor Inventory
TNF-α: Tumour necrosis factor α
IL-1β: Interleukin-1β
IL-2: Interleukin-2
IL-2R: Interleukin-2 receptor
IL-6: Interleukin-6
IL-10: Interleukin-10
IL-12: Interleukin-12
IL-13: Interleukin-13
CRP: C-reactive protein
ESR: Erythrocyte sedimentation rate
ACPA: Anti-citrullinated protein antibody
DAS 28: Disease Activity Score 28
SDAI: Simplified Disease Activity Index
CDAI: Clinical Disease Activity Index
FFbH: Funktionsfragebogen Hannover (Hannover Functional Ability Questionnaire)
VAS: Visual analogue scale
cs/tsDMARD: Conventional synthetic or targeted synthetic disease-modifying antirheumatic drugs
b/bsDMARD: Biological or biosimilar disease-modifying antirheumatic drugs
Na-EDTA: Sodium ethylenediaminetetraacetate
SCID-5: Structured Clinical Interview for DSM-5
LEDS: Life Events and Difficulties Schedule
OPD: Operationalized Psychodynamic Diagnosis
DIARI: Daily Inventory of Activity, Routine and Illness
IHI: Incidents and Hassles Inventory
BMI: Body mass index
ARIMA: Autoregressive integrated moving-average model
VAR: Vector autoregressive model
